# Increased expression of SKP2 is an independent predictor of locoregional recurrence in cervical cancer *via* promoting DNA-damage response after irradiation

**DOI:** 10.18632/oncotarget.10057

**Published:** 2016-06-15

**Authors:** Hung-Chun Fu, Yi-Chien Yang, Yun-Ju Chen, Hao Lin, Yu-Che Ou, Chan-Chao Chang Chien, Eng-Yen Huang, Hsuan-Ying Huang, Jui Lan, Hsi-Ping Chi, Ko-En Huang, Hong-Yo Kang

**Affiliations:** ^1^ Graduate Institute of Clinical Medical Sciences, Chang Gung University, College of Medicine, Kaohsiung, Taiwan; ^2^ Center for Menopause and Reproductive Medicine Research, Kaohsiung Chang Gung Memorial Hospital, Kaohsiung, Taiwan; ^3^ Department of Obstetrics and Gynecology, Kaohsiung Chang Gung Memorial Hospital and Chang Gung University College of Medicine, Kaohsiung, Taiwan; ^4^ Department of Dermatology, Kaohsiung Chang Gung Memorial Hospital and Chang Gung University College of Medicine, Kaohsiung, Taiwan; ^5^ Department of Radiation Oncology, Kaohsiung Chang Gung Memorial Hospital and Chang Gung University College of Medicine, Kaohsiung, Taiwan; ^6^ Department of Pathology, Kaohsiung Chang Gung Memorial Hospital and Chang Gung University College of Medicine, Kaohsiung, Taiwan; ^7^ Medical Sciences Division, University of Oxford, Oxford, England, UK

**Keywords:** SKP2, local recurrence, radioresistance, cervical cancer, DNA damage

## Abstract

Although radiation therapy was known to be effective to cervical cancer, loco-regional recurrences are frequently found in patients. We aimed to identify a molecular marker predicting the response of cervical cancer to radiotherapy. We included the patients (*n* = 149) with cervical cancer who had undergone radiotherapy from 2004 to 2006. Tumor samples were collected to examine the association between the expression of S-phase kinase-associated protein 2 (SKP2) and prognosis in cervical cancer. We found higher expression of SKP2 associated with recurrence (HRs: 2.52, *p* < 0.001), death (HRs: 2.01, *p* < 0.001) and higher locoregional recurrence rate (HRs: 3.76, *p* < 0.001). Cervical cancer cell lines with higher expression of SKP2 showed higher colony formation, cell survival rate and fewer DNA damages after irradiation. SKP2-C25, an inhibitor for SKP2 activity, dose-dependently decreased cell viability after irradiation and knockdown of SKP2 impaired DNA-damage response and sensitized the cervical cancer cells to irradiation. Our data showed the SKP2 represents a promising tool to identify patients with cervical cancer who have a higher risk of locoregional recurrence after radiotherapy. Targeting SKP2 may serve as a potential radiosensitizer for developing effective therapeutic strategies against cervical cancer.

## INTRODUCTION

Cervical carcinoma is the third common malignancy in women worldwide [1]. Radiation therapy with or without cisplatin-containing chemotherapy is the most commonly used treatment modality in patients with advanced cervical cancer. The treatment can achieve a 5-year overall survival rate of 67% [2]. Almost a half (44%) of the patients experience a recurrence and 35% of recurrent tumors occur loco-regionally [2]. Loco-regional recurrence (LRR) following radiotherapy is still a challenge because successful salvage treatment is difficult [3]. For patients with a high risk of LRR, surgical treatment after radiotherapy is considered for better local control and survival. However, the place and modality of surgery after radiotherapy continue to be debated [4–6].

Molecular profiling of tumor and biomarkers studies have attempted to predict the patients who are more likely to experience a recurrence after standard therapy and develop individualized treatments that improve survival rate. While the expression of EGFR, VEGF, COX-2, galectin-1, Sphingosine kinase 1 and tumor hypoxia may have a major impact on the survival of patients treated with definitive radiotherapy [3, 7–10]. The studies addressing on the molecular biomarkers predicting the locoregional recurrence in cervical cancer after radiotherapy need to be pursued to improve the clinical outcome.

S-phase kinase-associated protein 2 (SKP2) protein is an E3 ubiquitin ligase and a part of SKP1-Cul1-F-box (SCF) complexes. SKP2 is overexpressed and associated with poor prognosis in various human cancers [11]. Using genome-wide array comparative genomic hybridization, we previously profiled deoxyribonucleic acid (DNA) copy number alterations and identified *SKP*2 gene amplification confers tumor aggressiveness in myxofibrosarcoma [12]. Moreover, integrative genomics analysis [13, 14] and morphoproteomic evidence [15] also identified target over-expressed genes, including *SKP*2 gene of chromosome 5p gain in cervical cancer may contribute to cervical dysplastic lesion progression to invasive tumor. However, whether SKP2 play important roles on radiotherapy for cervical cancers remains obscure. Thus, our study aimed to (i) determine the immunohistochemical expression of SKP2 as a prognostic marker of patients with cervical cancer receiving radiotherapy, (ii) evaluate expression of SKP2 as a predictor of response to radiotherapy in a cohort of patients and cell lines, and (iii) investigate the role of SKP2 in response of DNA damage of cervical cancer cell lines.

## RESULTS

### Patient characteristics with SKP2 as an independent prognostic factor in cervical cancer

Between January 2004 and December 2006, there were 334 patients showed pathologic proof of cervical cancer. We enrolled 149 patients meeting the criteria of inclusion. The median follow-up duration was 73.1 months (range, 2–113 months). During the follow-up period, recurrence of disease and death were observed in 68 and 74 patients, respectively. In univariate analysis, several clinicopathologic variables displayed a relationship with disease-free survival (DFS) (Table 1). The recurrence of disease was statistically significantly associated with a number of clinicopathological variables including tumor stage (*P* < 0.001), histologic types (*p* = 0.008), SKP2 expression level (*p* < 0.001), serum squamous cell carcinoma (SCC) antigen level (*p* = 0.015) and carcinoembryonic antigen (CEA) level (*p* = 0.01) before treatment (Table 1). The SKP2 expression level before treatment showed the median percentage of positive cells was 9.0% (range, 0.4–60.3%; mean, 11.6%, Table 1). The mean level of SKP2 in recurrence group (17.2 ± 12.1%) was significantly higher than disease free group (6.8 ± 6.2%) (*P* < 0.001, Table 1). No correlation was observed with either age at diagnosis, histologic grades, tumor size, chemotherapy, radiotherapy or surgery pattern (Table 1). There were 35 patients (23.5%) received chemotherapy. Chemotherapy was not a significant factor of recurrence (*p* = 0.746, Table 1). Next, we performed a multivariate Cox regression analysis to assess associations of clinicopathological characteristics with recurrence of disease. As shown in Table 1, staining level of SKP2, histologic types, SCC level, CEA level, and FIGO stage were confirmed to be independent prognostic factors of recurrence in cervical cancer.

**Table 1:** Summary of patient distribution according to disease status

		Status of disease		Univariate	Multivariate
					Adjusted
Variable	Total	Recurrence	Disease free	*P* value	*P* value
**All cases**	149	68	77		
**Age**					
≥ 60	74 (49.7)	31 (43.7)	40 (56.3)		
< 60	75 (50.3)	37 (50.0)	37 (50.0)	0.445	0.724
**Stage (%)**					
lower (I,II)	120 (80.5)	46 (39.3)	71 (60.7)		
higher (III,IV)	29 (19.5)	22 (78.6)	6 (21.4)	< 0.001	0.027
**Histologic type (%)**					
squamous cell carcinoma	139 (93.3)	59 (43.7)	76 (56.3)		
adenocarcinoma	5 (3.4)	5 (100)	0		
adenosqumous cell carcinoma	5 (3.4)	4 (80.0)	1 (20.0)	0.008	0.001
**Histologic grade (%)**					
G1	10 (6.7)	5 (50.0)	5 (50.0)		
G2	37 (24.8)	11 (29.7)	26 (70.3)		
G3 and unknown	102 (68.5)	52 (53.1)	46 (46.9)	0.056	0.109
**Tumor size (%)**					
≥ 4 cm	81 (54.4)	43 (63.2)	38 (49.4)		
< 4 cm	68 (45.6)	25 (36.8)	39 (50.6)	0.098	0.214
**SKP2 expression level (SD)**	11.6 (10.6)	17.2 (12.1)	6.8 (6.2)	< 0.001	< 0.001
**SCC level (SD)**	12.5 (26.9)	18.6 (36.3)	6.8 (12.7)	0.015	0.042
**CEA level (SD)**	14.2 (57.4)	24.7 (82.4)	5.1 (11.7)	0.01	0.027
**Chemotherapy (%)**	35 (23.5%)	19 (54.3)	16 (45.7)	0.746	0.472
**Radiotherapy (%)**					
pelvic only	42 (28.1)	20 (30.8)	20 (28.6)		
pelvic and brachytherapy	97 (65.1)	45 (69.2)	50 (71.4)	0.606	0.428
dose (SD)	5955.0 (1559.1)	6045.2 (1702.8)	5895.1 (1431.5)	0.576	0.596
**Operation pattern (%)**					
no hysterectomy	105 (70.5)	54 (81.8)	51 (67.1)		
simple hysteretomy	6 (4.0)	0	6 (25.0)		
radical hysterectomy	31 (20.8)	12 (18.2)	19 (7.9))	0.030	0.703

Abbreviations: CEA: Carcinoembryonic antigen, G1: grade 1, G2: grade 2, G3: grade 3, SCC: squamous cell carcinoma antigen, SD: standard deviation.

### Expression of SKP2 presents as a novel biomarker in cervical cancer recurrence after radiation therapy

**Figure 1:**
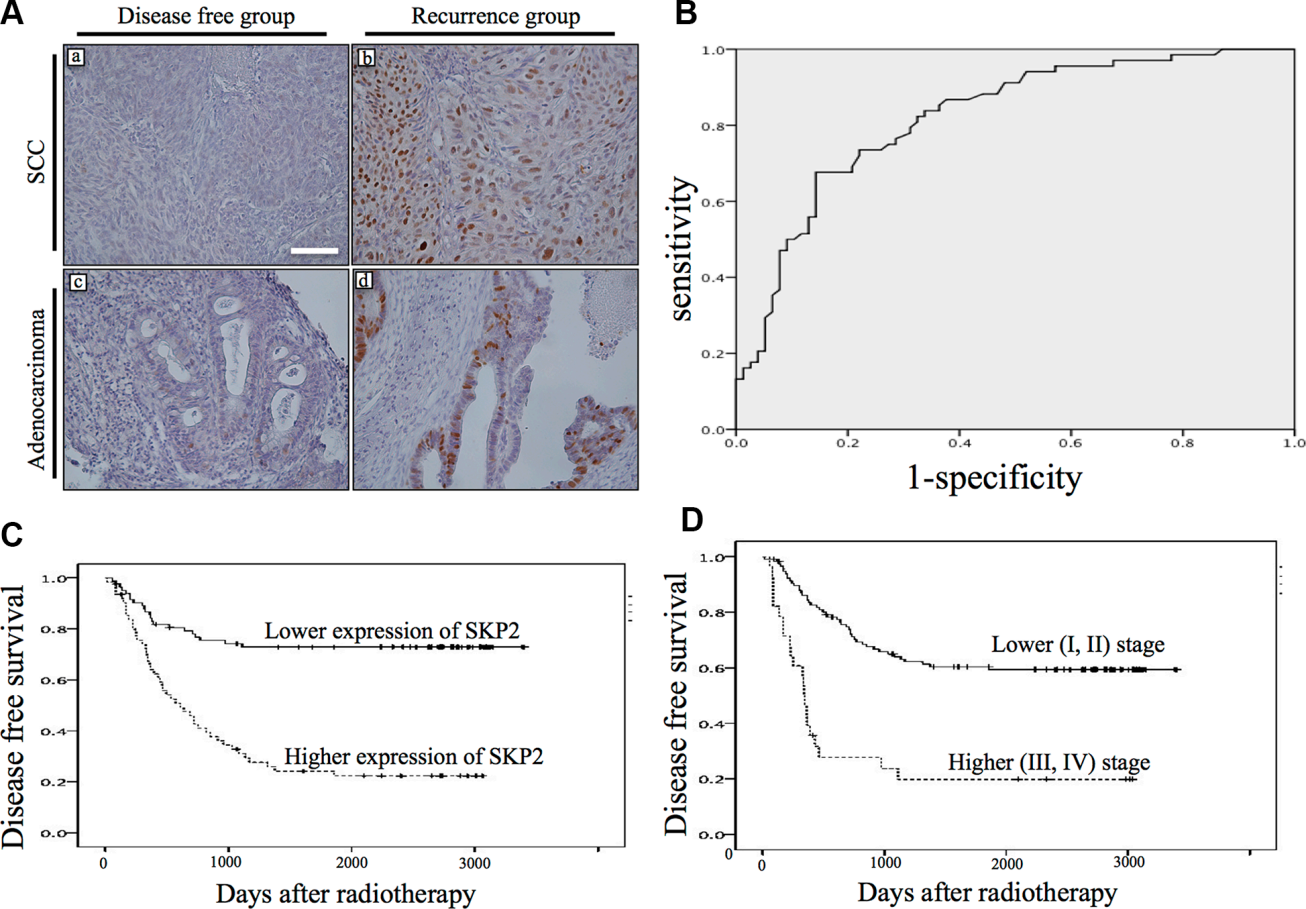
Immunostaining of SKP2 expression of pretreatment cervical cancer and the association with disease free survival (**A**) Weak (a, c) and strong (b, d) staining of SKP2 was shown in adenocarcinoma (bottom row) and squamous cell carcinoma (top row). (x200, size bar: 100 μm); (**B**) Receiver operator characteristic (ROC) curve was shown for expression of SKP2 in discriminating recurrence. Excellent accuracy was demonstrated (area under the curve, 0.82; standard error, 0.035). The cutoff level of SKP2 expression on the curve was 10.1% of nuclear staining. (**C**) SKP2 immunohistochemical staining expression status (higher: ≥ 10.1%, lower: < 10.1%) before radiotherapy exhibited a significant (log-rank, *p* < 0.001) relationship with patient disease free survival. (**D**) Kaplan-Meier survival curve for cervical cancer patients were classified as higher (III, IV) and lower (I, II) FIGO stage. FIGO stage status exhibited a significant (log-rank, *p* <0.001) relationship with patient disease free survival.

To investigate the role of SKP2 in human cervical cancer, the expression of SKP2 protein was evaluated by immunostaining in a series of 149 primary human cervical cancer specimens before treatment. SKP2 protein was shown in adenocarcinoma and squamous cell carcinoma of cervical cancer tissues. Only cells with a clear nuclear staining were scored as positive. SKP2 expression levels were higher in recurrence group (Figure 1A). Next, we investigated whether the expression level of SKP2 in cervical cancer tissue before treatment could be a biomarker of recurrence after radiotherapy. We performed the receiver operating characteristic (ROC) curve of SKP2 expression in cancer cells for recurrence of cancer analyzed in our study (Figure 1B), the area under the curve was 0.821, indicating very good discrimination. The threshold value of 10.1% was selected as the best cut-off score. The expression of tumor SKP2 could be categorized into lower expression (< 10.1 %) group and higher expression (≥ 10.1%) group. The Kaplan-Meier curves of DFS, which displayed a significant separation between the lower and higher expression of SKP2 groups of patients (*P* < 0.001 by log-rank test; Figure 1C). Expression of SKP2 of tumor specimen before treatment is a good prediction marker of recurrence of uteri cervical cancer after radiotherapy, as FIGO stage (*P* < 0.001; Figure 1D)

The expression patterns of SKP2 in cervical cancer tissues were correlated with clinicopathological variables including age at diagnosis, tumor stage, histological type, histological grade, tumor size, percentage of death and recurrence, recurrence site and the levels of SCC and CEA before therapy (Table 2). The high expression of SKP2 was found in 64 patients (43%), whereas low SKP2 expression was detected in 85 patients (57%) (Table 2). Patients with high expression level of SKP2 (≥ 10.1%) were associated with recurrence (hazard ratios (HRs): 2.52, 95% confidence interval (CI), 1.75–3.62, *P* < 0.001) and death (HRs: 2.01, 95% CI, 1.46–2.76, *P* < 0.001). Median DFS of patients whose tumors expressed high level of SKP2 (18.4 months; mean, 31.6 ± 31.4 months) was shorter compared with lower expression patients (83.9 months; mean, 65.5 ± 37.5 months), and this difference was significant (*P* < 0.001). Similarly, 46 (71.8%) of the 64 patients with high SKP2 expression of tumor and 28 (32.9%) of the 85 lower expression of tumor died of disease during the period of follow-up. This difference was significant (*P* < 0.001). Hence, recurrence, locoregional recurrence, death, larger tumor size, higher stage and poor differentiation occurred more frequently when cervical cancer in higher staining group for SKP2 (Table 2). Age, stage, histologic type, tumor size SCC level or CEA level was not associated with expression of SKP2 in the same subset of patients (Table 2). While FIGO stage was shown to be an important factor of recurrence of cervical cancer [7], here we further demonstrated that patients with tumors displaying high staining for SKP2 were more likely to recur at locoregional site (HRs: 3.76, CI, 1.91–7.16, *p* < 0.001) compared with patients who had lower SKP2 expression after radiation therapy.

**Table 2:** Demographic patient distribution according to SKP2 expression before therapy

		SKP2 expression score before therapy		
Variable	Total	≥ 10.1 %	< 10.1%	*P* value
**All cases**	149	64 (43.0)	85 (57.0)	
**Age (%)**				
≥ 60	74 (49.7)	29 (39.2)	45 (60.8)	
< 60	75 (50.3)	35 (46.7)	40 (53.3)	0.409
**Stage (%)**				
lower (1,2)	120 (80.5)	48 (40.0)	72 (60.0)	
higher (3,4)	29 (19.5)	16 (55.2)	13 (44.8)	0.029
**Histologic type (%)**				
squamous cell	139 (93.3)	58 (41.7)	81 (58.3)	
adenocarcinoma	5 (3,4)	3 (60.0)	2 (40.0)	
adenosqumous cell	5 (3.4)	3 (60.0)	2 (40.0)	0.54
**Histologic grade (%)**				
G1	10 (6.7)	5 (50.0)	5 (50.0)	
G2	37 (24.8)	8 (21.6)	29 (78.4)	
G3 and unknown	102 (68.5)	51 (50.0)	51 (50.0)	0.01
**Tumor size (%)**				
≥ 4 cm	81 (54.4)	41 (50.6)	40 (49.4)	
< 4 cm	68 (45.6)	23 (33.8)	45 (66.2)	0.047
**Death (%)**	74 (49.7)	46 (62.2)	28 (37.8)	< 0.001
**Recurrence (%)**	68 (46.9)	46 (67.6)	22 (32.4)	< 0.001
**Recurrence site (%)**				
locoregional	42 (61.8)	33 (71.7)	13 (28.3)	
distant	26 (38.2)	9 (40.9)	13 (59.1)	0.014
**SCC level before therapy (SD)**	12.4 (27.1)	11.9 (20.6)	13.0 (30.9)	0.315
**CEA level before therapy (SD)**	14.5 (58.2)	14.0 (10.4)	6.1 (3.0)	0.462

Abbreviations: CEA: Carcinoembryonic antigen, G1: grade 1, G2: grade 2, G3: grade 3, SCC: squamous cell carcinoma antigen, SD: standard deviation.

### SKP2 increases the survival of cervical cancer cells after irradiation

**Figure 2:**
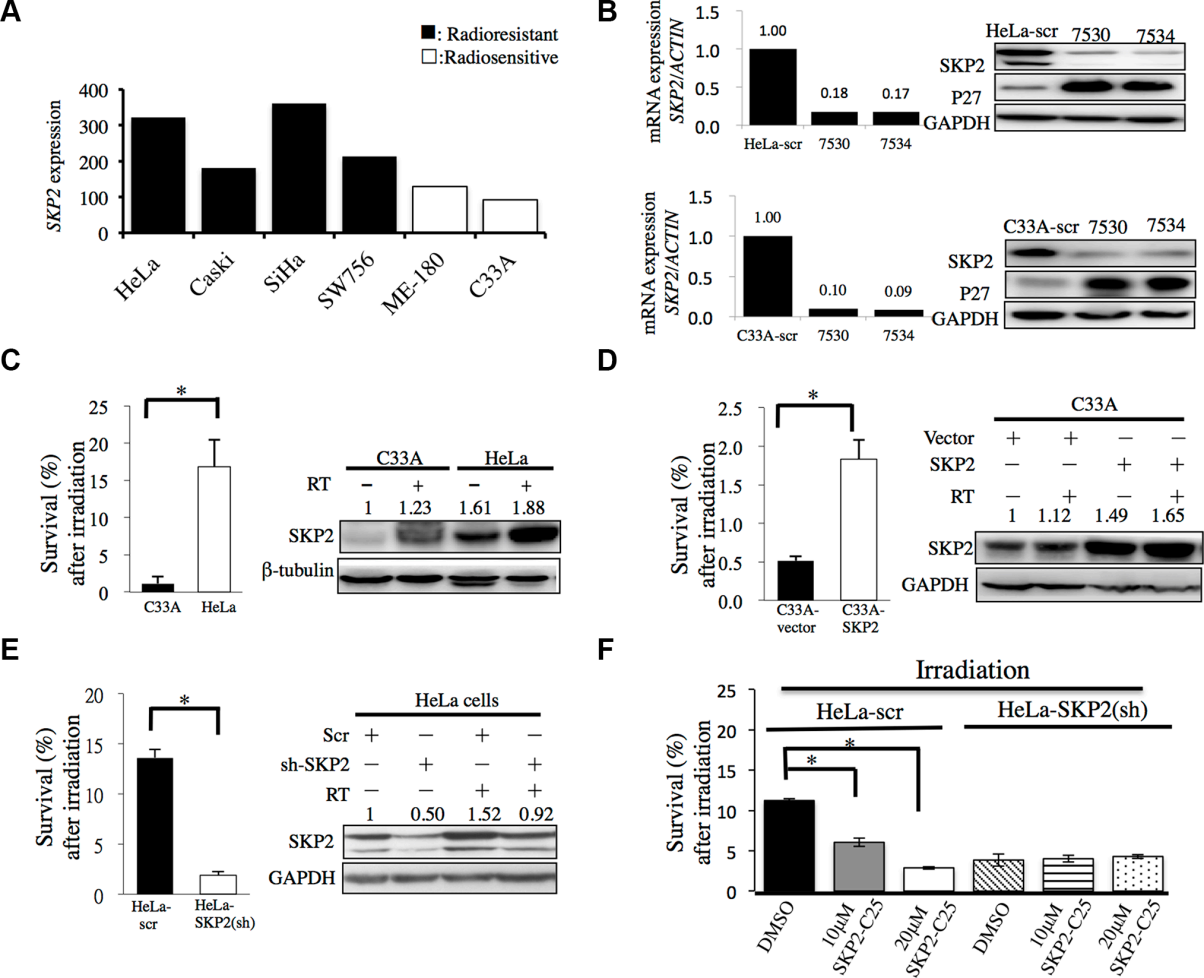
SKP2 enhanced cervical cancer cell survival upon ionizing radiation *in vitro* (**A**) *SKP*2 mRNA expressions of different cervical cancer cell lines were obtained from GEO Datasets database (GDS3233). The radioresistant cervical cell lines had significantly higher expression of SKP2 than radiosensitive cell lines (*P* = 0.007). (**B**) We decreased the expression of SKP2 of HeLa and C33A cell with lentivirus with pLKO.1-sh-SKP2 (TRCN0000007530 (7530) and TRCN0000007534 (7534)). Real time PCR and western blotting were done for evaluation of the efficiency of the lentivirus. (**C**) Parental human radioresistant HeLa and radiosensitive C33A cervical cancer cells, (**D**) C33A cells were overexpressed SKP2 by *SKP*2 cDNA transfected and compared with empty vectors control. (**E**) HeLa cells were decreased SKP2 by shRNA and compared with scrambled (Scr) RNA. (**F**) Control scramble shRNA or SKP2 shRNA-transfected HeLa cells were treated with the indicated doses of SKP2-C25 (SKP2 inhibitor). After irradiation, cell survival was detected by colony formation assay. Cell survival fraction was significantly reduced in dose-dependent manner in HeLa-scr cells treated with the indicated doses of SKP2-C25. However, The inhibitory effect of SKP2 inhibitor on cell survival was not seen in the cells when SKP2 was decreased. C-E. Clonogenic survival rates and expression of SKP2 were detected. Cells were irradiated (6 Gy) and clonogenic assay performed 14 days following irradiation. Clonogenic assays were performed during three independent experiments at each paired condition (with and without SKP2 modulation). (*P < 0.005).

To determine whether SKP2 is differentially expressed in radioresistant and radiosensitive cervical cell lines, microarray data of SKP2 mRNA expressions of different cervical cancer cell lines were obtained from GEO Datasets database (GDS3233) [16]. We found that SKP2 mRNA expressions were higher in radioresistant cervical cell lines (Figure 2A). To investigate the association of SKP2 expression with radioresistance, we decreased the expression of SKP2 with shRNA (TRCN0000007530 and TRCN0000007534) in HeLa and C33A cells (Figure 2B). Next, Western blotting analysis was performed to investigate the association between SKP2 protein expression and radiosensitivity in HeLa cells and C33A cells that were previously characterized as radioresistant and radiosensitive cervical cancer cells, respectively [17]. Indeed, we also noted a high protein expression of SKP2 in HeLa cells and a weak expression in C33A cells (Figure 2C). Therefore, we transfected the SKP2/scrambled shRNA in HeLa cells to knockdown SKP2 expression and stable clones were selected with puromycin (4μg/mL) (HeLa-scr/HeLa-SKP2(sh)). Empty plasmid or cDNA of SKP2 was transfected in C33A cells for SKP2 overexpression and stable clones were selected with zeocin (C33A-SKP2/C33A-vector). The expression level of SKP2 was confirmed by immunoblotting analysis. Transfection of cDNA with SKP2 increased the expression of SKP2 in C33A-SKP2 cells (Figure 2D). The shRNA of SKP2 decreased SKP2 expression in protein levels of HeLa-SKP2(sh) cells (Figure 2B, 2E). Furthermore, we assessed the effect of SKP2 in the radiation response of cervical cancer cells *in vitro*. The cell survival efficiency determined by colony-forming assay in C33A-SKP2/C33A-vector cells or HeLa-SKP2(sh)/scr cells. Demonstrated that SKP2-overexpressing C33A cells exhibited higher colony formation compared with vector control cells (Figure 2D). The survival fraction rate showed a statistically significantly suppressed in SKP2-knockdowned HeLa cells compared with control (Figure 2E). We further investigated whether inhibition of SKP2 activity decreased cervical cancer cells survival after irradiation by SKP2-C25, an inhibitor of SKP2 that has been shown to selectively suppress SKP2 E3 ligase activity [18]. As shown in Figure 2F, the survival fraction of HeLa-scr cells treated with SKP2-C25 was lower than DMSO group. Cell survival fraction was significantly reduced in dose-dependent manner in HeLa-scr cell line but not in SKP2-HeLa(sh) cells treated with the indicated doses of SKP2-C25 before irradiation. Together, these results suggest that SKP2 overexpressing cells showed increased cell survival and lower expression of SKP2 or loss of SKP2 activity in cervical cancer cells associated with lower survival fraction after irradiation.

### SKP2 enhances the responses of cervical cancer cells to DNA damage after irradiation

**Figure 3:**
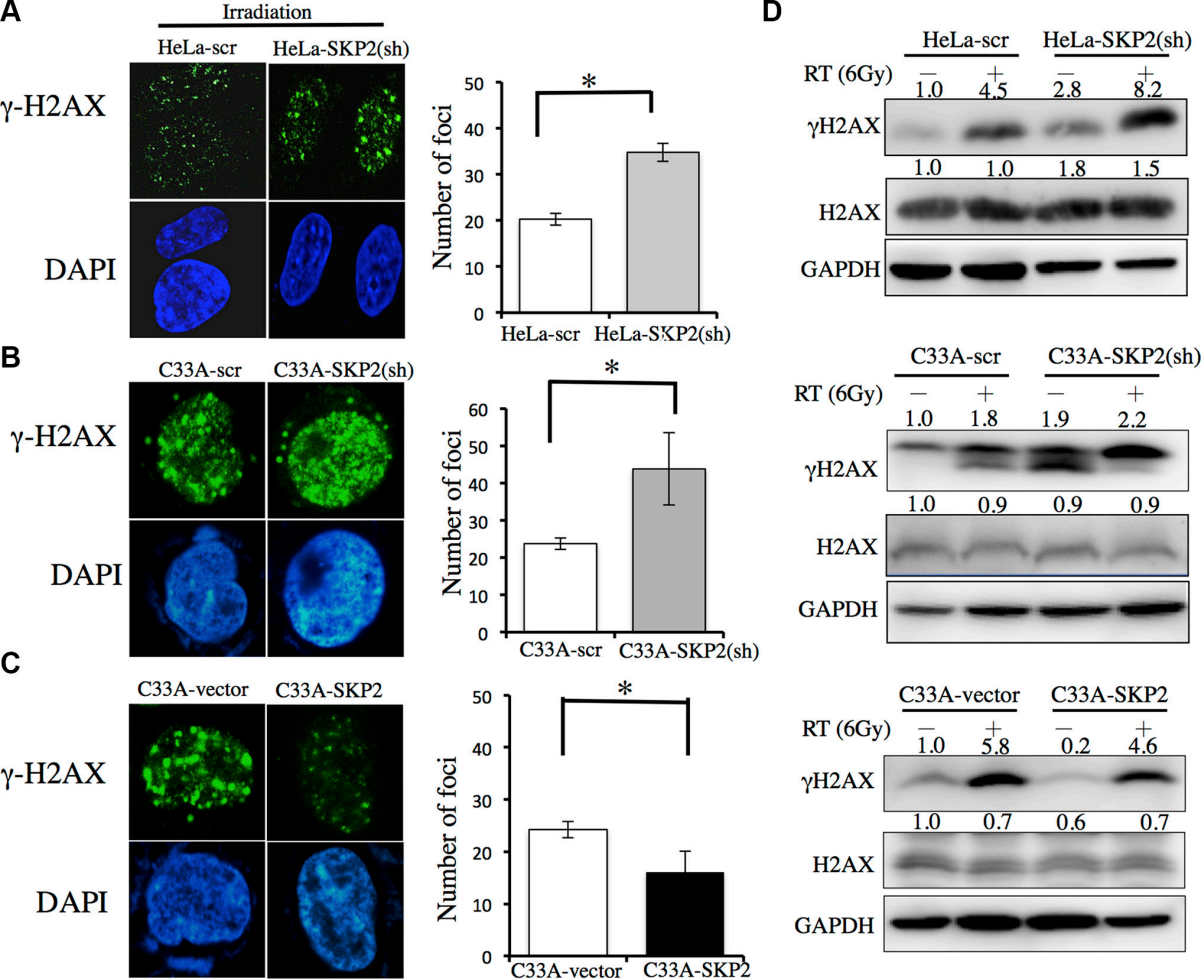
SKP2 increased DNA damages clearance upon ionizing radiation in human cervical cancer cell lines C33A-scr, C33A-SKP2(sh), C33A-vector, C33A-SKP2, HeLa-scr and HeLa-SKP2(sh) cells were treated with 6-Gy irradiation. After 4 hours incubated, immunofluorescence staining for γ-H2AX (green) and DNA counterstaining with DAPI (blue). Number of γ-H2AX was counted. Western blots were done to determine the level of γ-H2AX and H2AX. GAPDH was used as the protein-loading control. (**A**) More γ-H2AX foci were found in HeLa-SKP2(sh) than HeLa-scr cells, after irradiation. The results of western blotting showed greater expression in HeLa-SKP2(sh) than HeLa-scr cells following irradiation. (**B**) More γ-H2AX foci were found in C33A-SKP2(sh) than C33A-scr cells, after irradiation. The results of western blotting showed greater expression in C33A-SKP2(sh) than C33A-scr cells following irradiation. (**C**) More γ-H2AX foci were found in C33A-vector than C33A-SKP2 cells, after irradiation. (**D**) The results of western blotting showed, greater expression of γ-H2AX in HeLa-SKP2(sh), C33A-SKP2(sh) and C33A-vector cells than HeLa-scr, C33A-scr and C33A-SKP2 cells following irradiation, respectively. Lower expression SKP2 of cells associated higher expression of γ-H2AX after irradiation. (*P <0.001).

DNA damage clearance is a significant factor of radioresistance in cancer cells [19, 20] and γ-H2AX has been employed as a sensor of double-strained DNA breaks (DSBs) after γ-irradiation treatment [21]. Therefore, we investigated the effects of SKP2 expression on DNA damages after irradiation in HeLa and C33A cells which SKP2 expression was stably silenced or over-expressed. The immunofluorescence analysis revealed a much more γ-H2AX foci were noted in the nuclei of HeLa-SKP2(sh) cells than HeLa control cells (Figure 3A) (*P* < 0.001). More γ-H2AX foci localized in the nuclei of C33A-SKP2(sh) cells after exposure to irradiation than in control cells (Figure 3B) (*P* < 0.001). Significantly fewer γ-H2AX foci were noted in C33A-SKP2 cells than control cells (*P* < 0.001) (Figure 3C). The results of western blotting showed, greater expression of γ-H2AX in HeLa-SKP2(sh), C33A-SKP2(sh) and C33A-vector cells than HeLa-scr, C33A-scr and C33A-SKP2 cells following irradiation, respectively. Lower expression SKP2 of cells associated higher expression of γ-H2AX after irradiation (Figure 3D).

**Figure 4:**
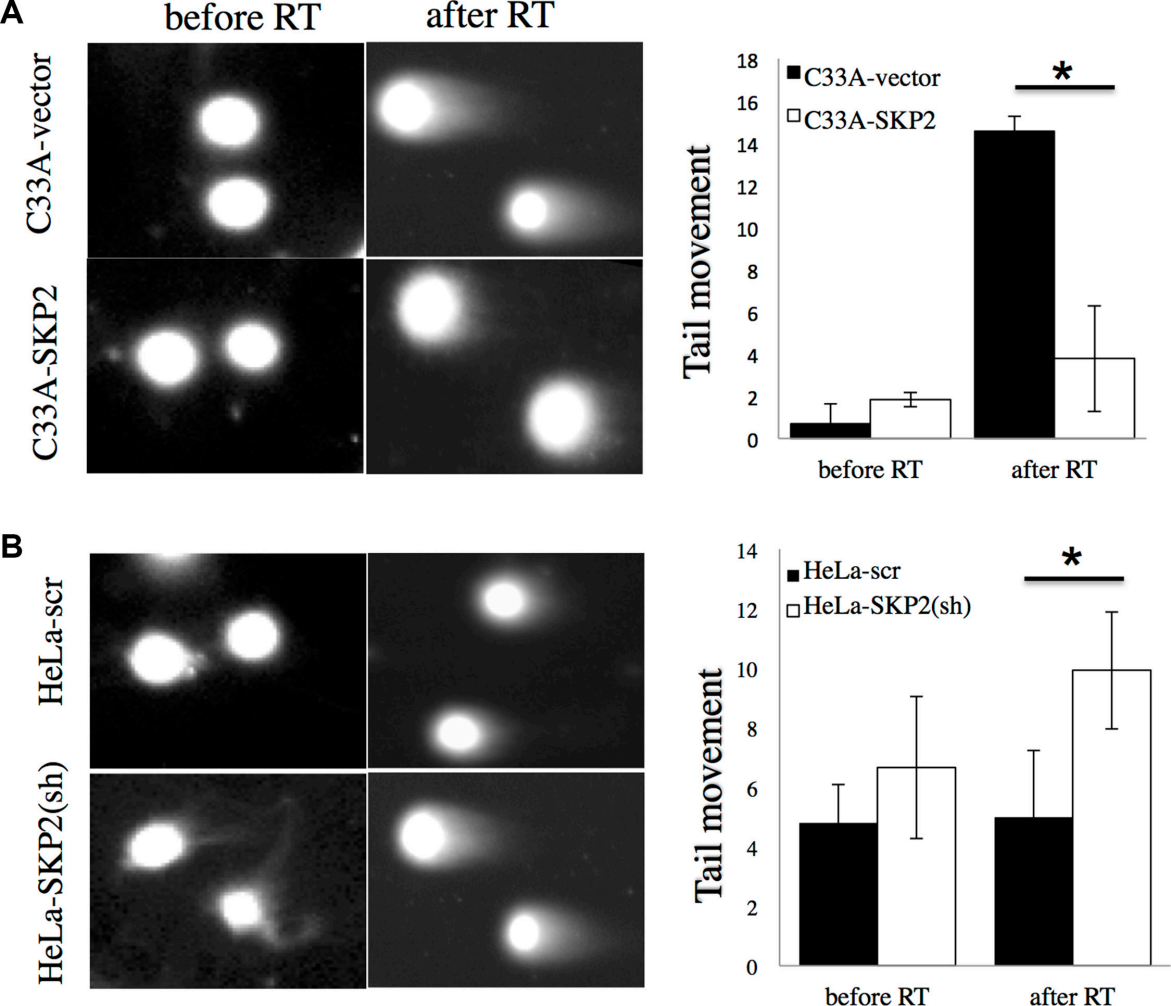
SKP2 enhances the responses of cervical cancer cells to DNA damage after irradiation Alkaline comet assay was performed to assess DNA damages after irradiation treatment (RT) 2 hours in (**A**) C33A-vector/C33A-SKP2 and (**B**) HeLa-scr /HeLa-SKP2(sh) stable clone cells. The assay was done in cells without irradiation (no RT) as control. Differences of tail movement before and after irradiation 2-hour were compared in (C33A-vector and C33A-SKP2) and (HeLa-scr and HeLa-SKP2(sh)). (*P < 0.001).

Since the single cell gel electrophoresis assay (comet assay) has been used as a useful reliable genotoxicity test that detects DNA migration caused by strand breaks [22], we applied the cells to be exposed to irradiation, allowed to recover for 2 hours, and then for detection of DNA damage by alkaline comet assay (Figure 4). Cells did not show significant different tail movement before irradiation. After receiving irradiation, C33A-vector cells produced a significant increase in the level tail moment than C33A-SKP2 cells (*P* < 0.05) (Figure 4A). While HeLa-scr did not show the significant difference of tail movement between before and after irradiation, increase of tail movement was observed in HeLa-SKP2(sh) after irradiation (Figure 4B). Together, these results suggested that SKP2 significantly increased the response of cervical cancer cells to efficiently repair DNA damage by irradiation.

### SKP2 promotes DNA double-strand breaks repair pathways

**Figure 5:**
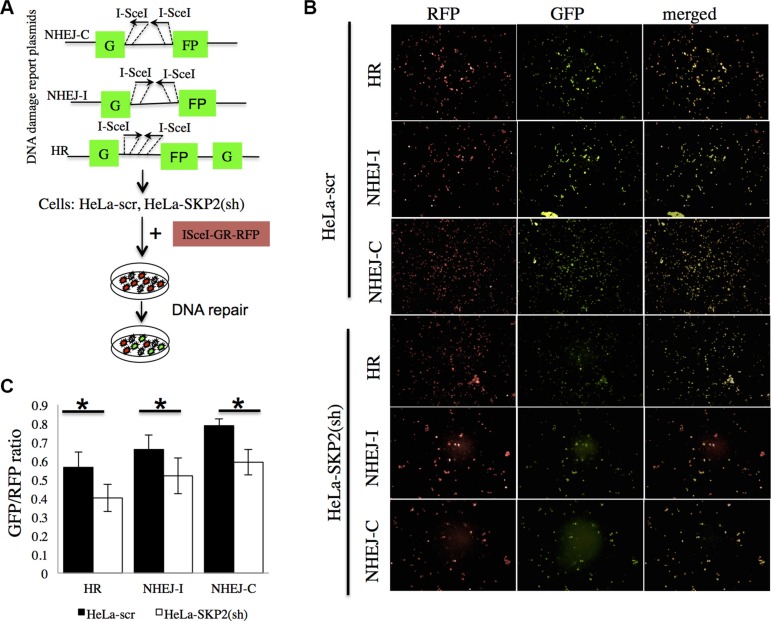
SKP2 increased the efficiency of NHEJ and HR repair of DSBs (**A**) The reporter plasmids of NHEJ-I, NHEJ-C and HR were used to generate the reporter cell lines with stable expression of the reporter. (**B**) The cells were transfected with the RFP–*ISce*I–GR showed RFP-positive signal. *ISce*I cut the reporter plasmid *ISce*I-sites and created DSBs of DNA. Cells that successfully repaired the DSBs showed GFP-positive signal. (**C**) The ration of GFP+/RFP+ presented the efficiency of DSBs repair with HR or NHEJ. HeLa-SKP2(sh) showed significantly lower efficiency of DSBs repair (both HR and NHEJ) than HeLa-scr. (*P < 0.005).

DNA double-strand breaks (DSBs) are critical events that can lead to cell death or genomic alterations including deletions, loss of heterozygosity, translocations, and chromosome loss. DSBs are repaired by non-homologous end-joining (NHEJ) and homologous recombination (HR), and dysfunction in these pathways result in genome instability and promote tumorigenesis [23]. To investigate the roles of SKP2 on DSB repair pathways in cervical cells after irradiation, we first generated reporter cell lines stably containing green fluorescent protein (GFP)-based reporter constructs. Induction of DSBs can be induced by the expression of *ISce*I and either NHEJ or HR pathway can repair DSBs and restore the function of the GFP gene [24]. The reporter plasmids of NHEJ-I, NHEJ-C or HR (Figure 5A) were individually transfected to indicated cell lines and the stable clones were selected under G418 (1 mg/ML). The stable clone cells were transfected with the RFP–*ISce*I–GR reported plasmid [25] to induce DSBs and showed RFP+ (Figure 5B). Cells that successfully repaired the DSBs showed GFP+ (Figure 5B). The quantitative GFP+/RFP+ ratio represented the efficiency of DSBs repair with HR or NHEJ (Figure 5C). HeLa cells with stable knockdown of SKP2 (HeLa-SKP2(sh)) showed significantly lower efficiency of DSBs repair regardless of whether of HR or NHEJ pathway (Figure 5C). These results suggested that SKP2 may improve the efficiency of DSBs repair, in both HR and NHEJ pathways.

## DISCUSSION

Chen TP, et al., reported higher expression of SKP2 in invasive cervical cancer than in cervical intraepithelial neoplasia and in normal cervical epithelium [26], it indicates that SKP2 may play important roles in cervical carcinogenesis and progression. In agreement with previous report [27], we found that a high expression of SKP2 in cervical cancer was correlated with higher stage, poorer differentiation, larger tumor size and deeper of invasion. Importantly, we identified a correlation between SKP2 expression, disease recurrence and decreased survival after radiotherapy that was not observed in previous report [27], which recruited patients who received surgical treatment, only 38 (11.4%) patients received postoperative radiation. The discrepancy could be in part due to the majority of patients in previous study enrolled 58 cases of cervical intraepithelial neoplasia III (CIN III), 102 cases of microinvasive SCC, 123 cases of invasive SCC, and most subjects were radiotherapy-naive. Conversely, the studied population in our present study was homogenous for cervical cancer patients who received the protocol of radiotherapy.

The important prognostic variables of cervical cancer are represented by FIGO stage, tumor size, depth of invasion, lymph-vascular space involvement and histological type [7]. Locoregional recurrence following radiotherapy is still a challenge because successful salvage treatment for LRR is difficult. Surgical treaments after radiotherapy expected some promising effects in reducing locoregional recurrence. However, clinical studies failed to establish a significant benefit [28–30], probably owing to the small number of patients studied and the lack of selection of patients with high risks of recurrence. While several biomarkers as prognosis factors for radiotherapy have been studied such as survivin, cIAP1 and Galectin-1 [3, 7, 31–35], the role of these molecules in concurrent chemoradiation therapy (CCRT) remains to be validated, because CCRT has been the gold standard treatment for locally advanced cervical cancer since 1999 [36]. Our study in a well-documented series of cervical cancer patients primarily treatment with radiotherapy reveals that higher SKP2 immunostaining is associated with poor DFS (*P* < 0.001), especially locoregional recurrence. In our series, FIGO stage of uteri cervical cancer is also an important variable of recurrence; patients with higher FIGO stage had higher recurrent rate and shorter DFS (Table 1 and Figure 1D). Higher stage was not an independent factor associated with locoregional recurrence. It meant response to radiotherapy was a different phenomenon not necessarily related to stage but to a variety of factors, such as cell biological factors [33]. While higher level of immunostaining of SKP2 has also been shown to be in poor response to radiotherapy in oral, nasopharyngeal and rectal cancers [37–39]. This study is the first to report that higher level of immunostaining of SKP2 predicts poor response to radiotherapy in cervical cancer. The results of this study also showed patients with high expression of SKP2 (> 10.1% nuclear stain) had higher recurrence of cancer after radiotherapy, especially locoregional recurrence (Table 2). The results implied the cervical cancer cells with high expression of SKP2 had high radioresistant ability.

To further extend our findings from the clinical data, *SKP*2 mRNA expressions between radiosensitive and radioresistant cervical cancer cell lines from GEO Datasets database (GDS3233) [16] were acquired to explore the correlation between *SKP*2 expression and radioresistance in cervical cancer cell lines. Indeed, we found that *SKP*2 mRNA expressions were higher in radioresistant cervical cell lines (Figure 2A). Expression of endogenous SKP2 was much weaker in C33A than HeLa cells (Figure 2). Previous study showed C33A cells were more radiosensitive than HeLa cells [17]. In our data, HeLa cells were more resistant to irradiation than C33A cells. Increased expression of SKP2 promoted the radiation resistance of C33A cells (Figure 2D). Decreased SKP2 level increased the radiation sensitivity of HeLa cells (Figure 2E). These data prove that SKP2 had a significant role in radioresistance. Increased SKP2 level could defend against radiation-induced cell death. The relation between SKP2 and response to radiotherapy might be due to the fact that SKP2 is involved in DNA double-strand break repair [40]. DNA repair is a significant factor of radioresistance and survival of irradiated tumor cells critically depends on DNA repair [41, 42]. SKP2 had been reported that play an important role in homologous recombination repair of DSBs through interaction with NBS1, a key component of the Mre11/Rad50/NBS1 (MRN) complex to recruit ATM to DNA foci for activation [40]. Our results also showed that SKP2 protein increased the ability of clearance of DSBs induced by irradiation in cervical cancer cell lines with decreasing Gamma-H2AX foci number and expression level in SKP2 overexpressing cells after irradiation. We hypothesize that SKP2 may interact with MRN associated protein complex and trigger ubiquitination of these associated proteins upon DSBs, which is critical for facilitating ATM recruitment for DNA damage repair and improve radioresistance of cervical cancer cells. Further studies are of interest to pursue this hypothesis.

Cisplatin, the most commonly used agent applied to CCRT in cervical cancers, cisplatin mechanism of action is that the drug induces its cytotoxic properties through binding to nuclear DNA and subsequent interference with normal transcription, and/or DNA replication mechanisms [43, 44]. Cisplatin has anti-neoplastic activity and radiosensitizer effects. However, administration of cisplatin may result in hematological, gastrointestinal, renal toxicity and neuropathy. Previous study showed inhibitor of SKP2 displayed potent effects to decrease cancer cell viability, but only slightly decreased cell viability of normal epithelial cells [18]. Due to the side effects of cisplatin, we aimed to investigate whether the SKP2 inhibitor may act as a radiosensitizer in cervical cancer, and with lower toxicity to normal cells. Our data showed the inhibitor of SKP2 (SKP2-C25) displayed potent effects to increase the radiosensitivity of HeLa cells (Figure 2F) suggesting that it may act as a potential radiosensitizer for cervical cancer. However, whether SKP2-C25 can work, as a better radiosensitizer with more specific anti-neoplastic effects and fewer side effects than cisplatin remains to be further investigated.

In conclusion, we found for the first time that high expression of SKP2 was correlated with poor response of radiotherapy and prognosis of cervical cancer. High expression of SKP2 of tumor predicts higher locoregional recurrence of cervical cancer after radiotherapy. The patients with high expression of SKP2 cervical cancer are at high risk of locoregional recurrence and might be appropriate for adjuvant hysterectomy after radiotherapy. These results provide insights into the role of SKP2 in cervical cancer and suggest that inhibitor of SKP2 is a good radiosensitizer with potential anti-neoplastic function that may act as novel experimental intervention for developing effective therapeutic strategies against cervical cancer.

## MATERIALS AND METHODS

### Patients

This is a retrospective study composed of patients treated between January 2004 and December 2006. Tissue samples from all patients showed pathologic proof of cervical cancer (total 334 patients). The stages were assigned according to the International Federation of Gynecology and Obstetrics (FIGO) 2009 system. The inclusion criteria were FIGO stage I–IV, the cell types (squamous, adenosquamous, or adenocarcinoma) and undergoing radiotherapy; this resulted of 202 patients. After excluding the patients, whose tissues were from outside the hospital, were insufficient, or did not receive regular follow-up, 149 patients were analyzed. All patients underwent external-beam RT to whole pelvis of 39.6-50.4 Gy, with a daily fraction of 1.8–2 Gy, 5 fractions per week. High-dose-rate intracavitary brachytherapy was delivered to 97 patients after the completion of external beam RT. The details of the RT have been described previously [3]. The cumulative biologically effective dose at point A was at least 70 Gy_10_. Most of them (109 patients) did not receive hysterectomy. Radical and simple hysterectomy were delivered to 31 and 6 patients, respectively. All patients underwent radiotherapy after biopsy of cervix uteri or hysterectomy. This study was approved by the Institutional Review Board in Kaohsiung Chang Gung Memorial Hospital (KCGMH) (102-0084C) and conducted in accordance with approved institutional guidelines. The clinic data were obtained as de-identified data released by KCGMH for research purpose. This study followed the requirement of reporting recommendations for tumor marker prognostic studies [45].

### Immunohistochemical staining

All tissues were obtained from formalin-fixed, paraffin-embedded tissue blocks. Representative are as of tumor were selected by a pathologist from hematoxylin stained sections. For immunohistochemical staining, sections were deparaffinized with xylene rinse, rehydrated with a graded alcohol series (100%, 95%, 85%, and 75%), and then rinsed with distilled water. Antigen retrieval was enhanced by citrate buffer (10 mM, pH 6.0). Endogenous peroxidase activity was quenched by incubation in 3% hydrogen peroxide solution. The slides were incubated with primary antibodies, SKP2 (Invitrogen, clone 2C8D9, 1:200) overnight at 4^°^C and further incubated with a secondary antibody 2 hours at 25^°^C. Antigen–antibody complexes were detected by DAB (Dako, Glostrup, Denmark) and counterstained with Gill's hematoxylin (Merck, Whitehouse, NJ, USA). The expression of SKP2 was considered positive if immunoreactivity was found in at nuclei. Digital image analysis was carried out to determine the nuclear staining levels by using “ImmunoRatio”, a web based analysis software [46]. Minimum of four microscopic field areas were analyzed for each tumor slide and the data were plotted as percent positive cells for SKP2 staining.

### Cell culture and reagents

Cervical cancer cell lines, HeLa and C33A cells were obtained from Bioresource Collection and Research Center (BCRC, Hsinchu, Taiwan) that cell lines authentication was performed by BCRC. Cells were cultured in Dulbecco's modified Eagle's medium (DMEM) containing 10% fetal bovine serum (FBS), 100 IU/mL penicillin, 100 mg/mL streptomycin and 0.4 mM L-glutamine (Sigma) in a humidified 95% atmosphere with 5% CO_2_ at 37^°^C and passaged for fewer than 6 months after receipt or resuscitation. SKP2-C25 (Xcessbio, San Diego, CA, USA) was prepared 10 mM stock solution in dimethyl sulfoxide.

### Transfection of cervical cancer cell lines

The SKP2 expression plasmid, pcDNA3–SKP2 was a gift of Dr. Hui-Kuan Lin [40]. For overexpression of the SKP2, C33A cells were transfected with pcDNA3–SKP2 or pcDNA3 using Lipofectamine 2000 reagent (Life Technologies, Carlsbad, CA, USA), according to the manufacturer's instructions. After transfection, the cells were grown in G418 at 800 μg/ml for stable clone selection. Selected stable clones (C33A-SKP2 and C33A-vector) were maintained in the growth medium with G418 at 500 μg/ml. The lentiviral vectors were obtained from Taiwan National RNAi Core Facility (Academia Sinica, Taiwan), including pLKO.1. null-T (5′-TCAGTTAACCACTTTTT-3′) and pLKO.1-sh-SKP2 (TRCN0000007530: 5′-GCCTAAGCT AAATCGAGAGAA-3′ and TRCN0000007534: 5′-GATA GTGTCATGCTAAAGAAT-3′). For viral infection, 3 × 10^4^ HeLa or C33A cells were incubated with lentivirus in the presence of 8 μg/ml polybrene, followed by 4 μg/ml puromycin selection for stable clones (C33A-SKP2(sh), C33A-scr, HeLa-SKP2(sh) and HeLa-scr) of lentivirus-transduced cells.

### Irradiation of cells

Cells were transferred to 25-cm2 flasks and incubated in DMEM with 10% FBS at 37°C with 5% CO_2_ for 24 hours. The flasks were placed on a linear accelerator Clinac 600C/D (VARIAN, USA) with a fixed source skin distance and X-ray irradiation at 4 Gy/min.

### Colony formation assay

HeLa or C33A cells were transfected with the SKP2, sh-SKP2 or the negative control (vector or scramble RNA), followed by selection for stable clones. After irradiation, 2000, 1000, and 500 cells were seeded separately in 6-well plates for colony formation. After 14 days, colonies were fixed and stained with a mixture of 6% glutaraldehyde and 0.5% crystal violet. Only if a single colony contained more than 50 cells was it scored using a microscope [47]. Each assay was performed in duplicate on three independent occasions.

### Immunoblotting

Cells were lysed in lysis buffer supplemented with 1% protease inhibitor cocktail (Roche Applied Science, Indianapolis, IN, USA). Proteins in whole-cell lysates were resolved by sodium dodecyl sulfate-polyacrylamide gel electrophoresis and transferred to polyvinylidene fluoride (PVDF) membranes. Membranes were probed with SKP2 (1:1000; Zymed), anti-γ-H2AX antibody (1:1000; Cell Signaling), H2AX (1:1000; Cell Signaling), beta-tubulin (1:500; Santa Cruz Biotechnology) or GAPDH (1:5,000; Chemicon) and then incubated with horseradish peroxidase-conjugated secondary antisera (Amersham, Buckinghamshire, UK). Enhanced chemiluminescence was performed with ECL-Plus (Amersham Pharmacia).

### Immunofluorescence staining of phospho-(Ser-139)- H2AX (γ-H2AX)

Cells with/without irradiation were transferred to slides at a density of 3 × 10^3^ cells using cytospin. Immunofluorescence staining of γ-H2AX was performed as previously described [48]. Briefly, proteins were immunolabeled by incubating with anti-γ-H2AX antibody (1:200; Cell Signaling) and goat anti-rabbit Alexa Fluor 568- conjugated antibody (1:500; Invitrogen). Slides were examined under a Nikon E800 microscope, and images were collected using a SPOT RT3 microscope camera. Captured images were analyzed for foci using AlphaEase FC software, and foci were quantified as described previously [49].

### Alkaline comet assay

Indicated cells were harvested and washed with ice-cold phosphate-buffered saline (PBS) before and 2 hours after irradiation. Alkaline comet assay was performed as previous described [44, 50]. Briefly, the cell suspension (1 × 10^5^ cells/ml) was mixed with low melting point agarose (Sigma) at a final concentration of 1% and cast on microscopic slides. Approximately 50 μL of the mixture of agarose and cells were placed on slides, and the agarose was solidified at 4°C for 45 min. After solidification, the cover slips were removed and the slides placed in the lysing solution (2.5 M NaCl, 100 mM Na_2_EDTA, 10 mM Tris, pH 10 and 1% Triton X-100) for 1 h at 4°C in the dark. Later, the slides were placed in a horizontal gel electrophoresis unit filled with fresh electrophoretic buffer (1 mM Na_2_EDTA and 300 mM NaOH, pH 13) and left in this buffer for 40 min for DNA unwinding. Without changing the alkali solution the slides were electrophoresed for 30 min at 25 V (1.2 V/cm, 42–44 mA) at 8°C. After electrophoresis, the slides were rinsed with 0.4 M Tris, pH 7.5 (3 × 5 min) and then stained with 1 μM 4′,6-diamidine-2-phenylindole dihydrochloride (DAPI, 50 μl per slide).

### NHEJ and HR assay

NHEJ and HR assay was modified the method as previous described [24]. Briefly, we generated reporter cell lines, indicated cells were transfected with NHEJ-I, NHEJ-C, or HR reporter plasmid (kind gift of Prof. Vera Gorbunova) [24]. G418, at 1mg/ml, was added to the media 1 day post-transfection. Colonies were picked after 7–10 days on selection. Transfection with the RFP–*ISce*I–GR (from Addgene) was performed. The final concentration of triamcinolone acetonide (Sigma, St Louis, MO) added was 10^−7^ M [25]. After adding the triamcinolone acetonide, RFP–*ISce*I–GR chimera protein shifted to nuclear and cut the *ISce*I site of reporter plasmid. Cells were harvested and washed with cold PBS after adding the triamcinolone acetonide 2 hours. After fixation in 4% buffered paraformaldehyde in PBS for 10 min at room temperature, slides were examined under a Nikon E800 microscope, and images were collected using a SPOT RT3 microscope camera. Captured images were analyzed for foci of GFP and RFP. The numbers of GFP+ and RFP+ cells were determined. The ratio between GFP+ and RFP+ cells was used as a measure of DSB repair efficiency.

### Statistical analysis

Comparisons between SKP2 high-expression and low-expression groups for clinicopathologic factors were evaluated using the *x*^2^ with Fisher exact. The median follow-up duration was 73.1 months (range, 2–113 months) for 149 independently validated cases. The endpoint analyzed was disease-free survival (DFS), last visit or death. The durations were calculated from the date of operation until the occurrence of recurrence or last follow-up appointment. Univariate survival analyses were conducted using Kaplan–Meier plots and the statistical significance of difference between curves was evaluated by the two-sided log-rank test. Furthermore, hazard ratios (HRs) and 95% confidence intervals (CIs), which were computed from univariate and multivariable Cox proportional hazards regression models were used to assess associations between SKP2, recurrence and survival. The two-sided Student *t*-test was used to analyze clinicopathologic factors, colony formation assay, γ-H2AX foci, tail movement and ratio of GFP+/RFP+ for cell line samples. All of the statistical analyses were performed using the SPSS statistical package. All *P* values less than .05 were considered statistically significant.
