# Correction: Increased expression of SKP2 is an independent predictor of locoregional recurrence in cervical cancer via promoting DNA-damage response after irradiation

**DOI:** 10.18632/oncotarget.20269

**Published:** 2017-08-14

**Authors:** Hung-Chun Fu, Yi-Chien Yang, Yun-Ju Chen, Hao Lin, Yu-Che Ou, Chan-Chao Chang Chien, Eng-Yen Huang, Hsuan-Ying Huang, Jui Lan, Hsi-Ping Chi, Ko-En Huang, Hong-Yo Kang

**Present:** The grant funding number is incorrect.

**Correct:** The proper number is CMRPD891183. The authors sincerely apologize for this error.

Original article: Oncotarget. 2016; 7:44047-44061. https://doi.org/10.18632/oncotarget.10057

